# 5-(2-Fluoro-4-nitro­phen­yl)-1,3,4-thia­diazole-2-amine

**DOI:** 10.1107/S1600536809016614

**Published:** 2009-05-14

**Authors:** Yao Wang, Rong Wan, Feng Han, Peng Wang

**Affiliations:** a Department of Applied Chemistry, College of Science, Nanjing University of Technology, No.5 Xinmofan Road, Nanjing, Nanjing 210009, People’s Republic of China

## Abstract

The title compound, C_8_H_5_FN_4_O_2_S, was synthesized by the reaction of 2-fluoro-4-nitro­benzoic acid and thio­semicarbazide. The dihedral angle between the thia­diazole and benzene rings is 27.1 (2)°. In the crystal, inter­molecular N—H⋯N and C—H⋯O hydrogen bonds link the mol­ecules.

## Related literature

For general background to the biological activity of 1,3,4-thia­diazole derivatives, see: Nakagawa *et al.* (1996 ▶); Wang *et al.* (1999 ▶). For bond-length data, see: Allen *et al.* (1987 ▶).
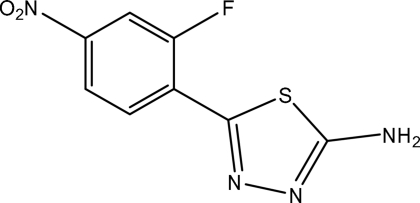


## Experimental

### 

#### Crystal data

C_8_H_5_FN_4_O_2_S
*M* *_r_* = 240.22
Monoclinic, 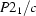

*a* = 9.1780 (18) Å
*b* = 9.3720 (19) Å
*c* = 11.413 (2) Å
β = 102.55 (3)°
*V* = 958.2 (3) Å^3^
*Z* = 4
Mo *K*α radiation
μ = 0.34 mm^−1^
*T* = 293 K
0.20 × 0.10 × 0.10 mm

#### Data collection

Enraf–Nonius CAD-4 diffractometer
Absorption correction: ψ scan (North *et al.*, 1968 ▶) *T*_min_ = 0.935, *T*_max_ = 0.967
1832 measured reflections
1720 independent reflections
1301 reflections with *I* > 2σ(*I*)
*R*_int_ = 0.047
3 standard reflections every 200 reflections intensity decay: 1%

#### Refinement

*R*[*F* ^2^ > 2σ(*F* ^2^)] = 0.056
*wR*(*F* ^2^) = 0.194
*S* = 1.01
1720 reflections
145 parameters
H-atom parameters constrained
Δρ_max_ = 0.43 e Å^−3^
Δρ_min_ = −0.47 e Å^−3^

### 

Data collection: *CAD-4 Software* (Enraf–Nonius, 1989 ▶); cell refinement: *CAD-4 Software*; data reduction: *XCAD4* (Harms & Wocadlo,1995 ▶); program(s) used to solve structure: *SHELXS97* (Sheldrick, 2008 ▶); program(s) used to refine structure: *SHELXL97* (Sheldrick, 2008 ▶); molecular graphics: *SHELXTL* (Sheldrick, 2008 ▶); software used to prepare material for publication: *SHELXL97*.

## Supplementary Material

Crystal structure: contains datablocks global, I. DOI: 10.1107/S1600536809016614/at2775sup1.cif

Structure factors: contains datablocks I. DOI: 10.1107/S1600536809016614/at2775Isup2.hkl

Additional supplementary materials:  crystallographic information; 3D view; checkCIF report

## Figures and Tables

**Table 1:** Hydrogen-bond geometry (Å, °)

*D*—H⋯*A*	*D*—H	H⋯*A*	*D*⋯*A*	*D*—H⋯*A*
N4—H4*A*⋯N3^i^	0.86	2.16	3.000 (5)	167
C3—H3*B*⋯O1^ii^	0.93	2.54	3.182 (7)	126

Symmetry codes: (i) 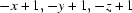; (ii) 
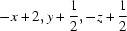
.

**Fig. 1.**
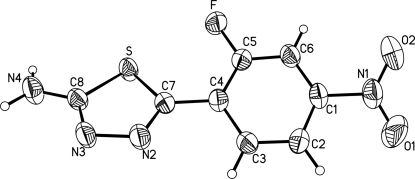
A view of the molecular structure of (I). Displacement ellipsoids are drawn at the 50% probability level.

**Fig. 2.**
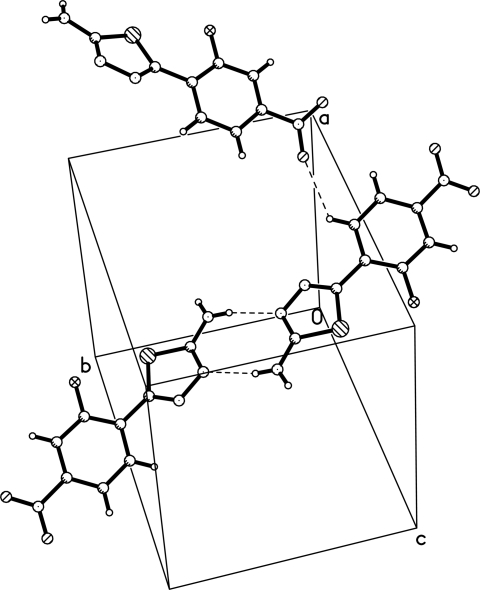
Partial packing view showing the hydrogen-bonded network. Dashed lines indicate intermolecular N—H···N and C—H···O hydrogen bonds.

## supplementary crystallographic information

### Comment

1,3,4-Thiadiazole derivatives represent an interesting class of compounds possessing broad spectrum biological activities (Nakagawa *et al.*, 1996). These compounds are known to exhibit diverse biological effects, such as insecticidal, fungicidal activities (Wang *et al.*, 1999).

The structure of the title compound, (I), is shown in Fig. 1, in which the bond lengths and angles are generally within normal ranges (Allen *et al.*, 1987). The dihedral angle between the thiadiazole and benzene ring is 27.1 (2)°. There are intermolecular N—H···N and C—H···O hydrogen bonds, linking the molecules, forming chains along the *b* axis (Fig. 2),.

### Experimental

2-Fluoro-4-nitrobenzoic acid (2 mmol) and thiosemicarbazide (5 mmol) were mixed in a 25 ml flask, and kept in the oil bath at 363 K for 6 h. After cooling, the crude product (I) precipitated and was filted. Pure compound (I) was obtained by crystallization from ethanol (20 ml). Crystals of (I) suitable for X-ray diffraction were obtained by slow evaporation of an acetone solution.

### Refinement

H atoms were placed geometrically with C—H = 0.93 Å and N—H = 0.86 Å, and included in the refinement in riding motion approximation with *U*_iso_(H) = 1.2 or 1.5*U*_eq_ of the carrier atom.

### Crystal data

**Table:**

C_8_H_5_FN_4_O_2_S	*F*(000) = 488
*M**_r_* = 240.22	*D*_x_ = 1.665 Mg m^−^^3^
Monoclinic, *P*2_1_/*c*	Melting point: 476 K
Hall symbol: -P 2ybc	Mo *K*α radiation, λ = 0.71073 Å
*a* = 9.1780 (18) Å	Cell parameters from 25 reflections
*b* = 9.3720 (19) Å	θ = 9–12°
*c* = 11.413 (2) Å	µ = 0.34 mm^−^^1^
β = 102.55 (3)°	*T* = 293 K
*V* = 958.2 (3) Å^3^	Block, colourless
*Z* = 4	0.20 × 0.10 × 0.10 mm

### Data collection

**Table:**

Enraf–Nonius CAD-4 diffractometer	1301 reflections with *I* > 2σ(*I*)
Radiation source: fine-focus sealed tube	*R*_int_ = 0.047
graphite	θ_max_ = 25.2°, θ_min_ = 2.3°
ω/2θ scans	*h* = 0→11
Absorption correction: ψ scan (North *et al.*, 1968)	*k* = 0→11
*T*_min_ = 0.935, *T*_max_ = 0.967	*l* = −13→13
1832 measured reflections	3 standard reflections every 200 reflections
1720 independent reflections	intensity decay: 1%

### Refinement

**Table:**

Refinement on *F*^2^	Primary atom site location: structure-invariant direct methods
Least-squares matrix: full	Secondary atom site location: difference Fourier map
*R*[*F*^2^ > 2σ(*F*^2^)] = 0.056	Hydrogen site location: inferred from neighbouring sites
*wR*(*F*^2^) = 0.194	H-atom parameters constrained
*S* = 1.01	*w* = 1/[σ^2^(*F*_o_^2^) + (0.1*P*)^2^ + 2.*P*] where *P* = (*F*_o_^2^ + 2*F*_c_^2^)/3
1720 reflections	(Δ/σ)_max_ < 0.001
145 parameters	Δρ_max_ = 0.43 e Å^−^^3^
0 restraints	Δρ_min_ = −0.47 e Å^−^^3^

### Special details

**Table:**

Geometry. All e.s.d.'s (except the e.s.d. in the dihedral angle between two l.s. planes) are estimated using the full covariance matrix. The cell e.s.d.'s are taken into account individually in the estimation of e.s.d.'s in distances, angles and torsion angles; correlations between e.s.d.'s in cell parameters are only used when they are defined by crystal symmetry. An approximate (isotropic) treatment of cell e.s.d.'s is used for estimating e.s.d.'s involving l.s. planes.
Refinement. Refinement of *F*^2^ against ALL reflections. The weighted *R*-factor *wR* and goodness of fit *S* are based on *F*^2^, conventional *R*-factors *R* are based on *F*, with *F* set to zero for negative *F*^2^. The threshold expression of *F*^2^ > σ(*F*^2^) is used only for calculating *R*-factors(gt) *etc*. and is not relevant to the choice of reflections for refinement. *R*-factors based on *F*^2^ are statistically about twice as large as those based on *F*, and *R*- factors based on ALL data will be even larger.

### Fractional atomic coordinates and isotropic or equivalent isotropic displacement parameters (Å^2^)

**Table:**

	*x*	*y*	*z*	*U*_iso_*/*U*_eq_	
S	0.65883 (14)	0.15613 (12)	0.66472 (10)	0.0442 (4)	
F	0.6863 (3)	−0.1515 (3)	0.6466 (2)	0.0539 (8)	
N1	0.9259 (5)	−0.4021 (5)	0.3718 (4)	0.0537 (11)	
C1	0.8706 (5)	−0.2697 (4)	0.4162 (4)	0.0404 (10)	
O1	1.0171 (6)	−0.3935 (5)	0.3093 (4)	0.0892 (15)	
O2	0.8765 (4)	−0.5153 (4)	0.4009 (4)	0.0656 (11)	
N2	0.6452 (5)	0.2128 (4)	0.4420 (3)	0.0449 (9)	
C2	0.8888 (5)	−0.1427 (5)	0.3565 (4)	0.0448 (11)	
H2B	0.9387	−0.1415	0.2938	0.054*	
N3	0.5854 (5)	0.3311 (4)	0.4867 (3)	0.0475 (10)	
C3	0.8298 (5)	−0.0180 (5)	0.3938 (4)	0.0412 (10)	
H3B	0.8386	0.0674	0.3544	0.049*	
N4	0.5320 (5)	0.4162 (4)	0.6663 (3)	0.0535 (11)	
H4A	0.4951	0.4943	0.6329	0.064*	
H4B	0.5347	0.4016	0.7412	0.064*	
C4	0.7568 (5)	−0.0197 (4)	0.4903 (4)	0.0358 (10)	
C5	0.7505 (5)	−0.1484 (5)	0.5495 (4)	0.0388 (10)	
C6	0.8026 (5)	−0.2754 (5)	0.5131 (4)	0.0421 (10)	
H6A	0.7925	−0.3609	0.5519	0.050*	
C7	0.6885 (5)	0.1134 (4)	0.5224 (4)	0.0367 (10)	
C8	0.5851 (5)	0.3175 (4)	0.6019 (4)	0.0378 (10)	

### Atomic displacement parameters (Å^2^)

**Table:**

	*U*^11^	*U*^22^	*U*^33^	*U*^12^	*U*^13^	*U*^23^
S	0.0663 (8)	0.0343 (6)	0.0343 (6)	0.0124 (5)	0.0166 (5)	0.0053 (4)
F	0.0753 (19)	0.0440 (16)	0.0517 (16)	0.0078 (13)	0.0340 (14)	0.0074 (12)
N1	0.069 (3)	0.038 (2)	0.054 (2)	0.013 (2)	0.012 (2)	−0.0076 (19)
C1	0.047 (2)	0.031 (2)	0.042 (2)	0.0064 (19)	0.0066 (19)	−0.0057 (19)
O1	0.125 (4)	0.067 (3)	0.096 (3)	0.032 (3)	0.068 (3)	−0.003 (2)
O2	0.075 (3)	0.041 (2)	0.075 (3)	0.0038 (18)	0.003 (2)	−0.0161 (19)
N2	0.068 (3)	0.0328 (19)	0.0379 (19)	0.0120 (18)	0.0200 (18)	0.0030 (16)
C2	0.053 (3)	0.040 (3)	0.045 (2)	0.006 (2)	0.019 (2)	−0.005 (2)
N3	0.073 (3)	0.0292 (19)	0.045 (2)	0.0150 (18)	0.0237 (19)	0.0044 (16)
C3	0.055 (3)	0.032 (2)	0.038 (2)	0.0018 (19)	0.014 (2)	0.0020 (18)
N4	0.084 (3)	0.040 (2)	0.039 (2)	0.020 (2)	0.018 (2)	0.0030 (17)
C4	0.040 (2)	0.032 (2)	0.034 (2)	0.0034 (18)	0.0068 (18)	−0.0020 (17)
C5	0.043 (2)	0.037 (2)	0.038 (2)	0.0015 (19)	0.0131 (18)	−0.0014 (18)
C6	0.056 (3)	0.030 (2)	0.041 (2)	0.003 (2)	0.012 (2)	0.0016 (18)
C7	0.042 (2)	0.031 (2)	0.038 (2)	0.0026 (18)	0.0120 (19)	0.0016 (18)
C8	0.048 (2)	0.032 (2)	0.035 (2)	0.0058 (18)	0.0101 (19)	0.0053 (17)

### Geometric parameters (Å, °)

**Table:**

S—C8	1.746 (4)	C2—H2B	0.9300
S—C7	1.750 (4)	N3—C8	1.321 (6)
F—C5	1.363 (5)	C3—C4	1.409 (6)
N1—O1	1.214 (6)	C3—H3B	0.9300
N1—O2	1.227 (5)	N4—C8	1.338 (6)
N1—C1	1.472 (6)	N4—H4A	0.8600
C1—C6	1.384 (6)	N4—H4B	0.8600
C1—C2	1.400 (6)	C4—C5	1.389 (6)
N2—C7	1.308 (5)	C4—C7	1.478 (6)
N2—N3	1.382 (5)	C5—C6	1.381 (6)
C2—C3	1.392 (6)	C6—H6A	0.9300
			
C8—S—C7	86.65 (19)	H4A—N4—H4B	120.0
O1—N1—O2	124.0 (4)	C5—C4—C3	117.9 (4)
O1—N1—C1	118.6 (4)	C5—C4—C7	123.2 (4)
O2—N1—C1	117.4 (4)	C3—C4—C7	118.9 (4)
C6—C1—C2	123.0 (4)	F—C5—C6	117.7 (4)
C6—C1—N1	119.4 (4)	F—C5—C4	119.0 (4)
C2—C1—N1	117.6 (4)	C6—C5—C4	123.3 (4)
C7—N2—N3	113.4 (3)	C5—C6—C1	116.9 (4)
C3—C2—C1	118.0 (4)	C5—C6—H6A	121.5
C3—C2—H2B	121.0	C1—C6—H6A	121.5
C1—C2—H2B	121.0	N2—C7—C4	120.6 (4)
C8—N3—N2	112.1 (3)	N2—C7—S	113.8 (3)
C2—C3—C4	120.8 (4)	C4—C7—S	125.7 (3)
C2—C3—H3B	119.6	N3—C8—N4	124.0 (4)
C4—C3—H3B	119.6	N3—C8—S	114.1 (3)
C8—N4—H4A	120.0	N4—C8—S	121.9 (3)
C8—N4—H4B	120.0		
			
O1—N1—C1—C6	−161.4 (5)	C4—C5—C6—C1	3.3 (7)
O2—N1—C1—C6	18.5 (6)	C2—C1—C6—C5	0.5 (7)
O1—N1—C1—C2	19.2 (7)	N1—C1—C6—C5	−178.9 (4)
O2—N1—C1—C2	−161.0 (5)	N3—N2—C7—C4	−179.2 (4)
C6—C1—C2—C3	−2.8 (7)	N3—N2—C7—S	0.2 (5)
N1—C1—C2—C3	176.7 (4)	C5—C4—C7—N2	−153.1 (4)
C7—N2—N3—C8	0.0 (6)	C3—C4—C7—N2	25.6 (6)
C1—C2—C3—C4	1.3 (7)	C5—C4—C7—S	27.5 (6)
C2—C3—C4—C5	2.2 (7)	C3—C4—C7—S	−153.7 (4)
C2—C3—C4—C7	−176.6 (4)	C8—S—C7—N2	−0.2 (4)
C3—C4—C5—F	177.2 (4)	C8—S—C7—C4	179.2 (4)
C7—C4—C5—F	−4.0 (7)	N2—N3—C8—N4	−179.3 (4)
C3—C4—C5—C6	−4.7 (7)	N2—N3—C8—S	−0.2 (5)
C7—C4—C5—C6	174.1 (4)	C7—S—C8—N3	0.2 (4)
F—C5—C6—C1	−178.5 (4)	C7—S—C8—N4	179.4 (4)

### Hydrogen-bond geometry (Å, °)

**Table:**

*D*—H···*A*	*D*—H	H···*A*	*D*···*A*	*D*—H···*A*
N4—H4A···N3^i^	0.86	2.16	3.000 (5)	167
C3—H3B···O1^ii^	0.93	2.54	3.182 (7)	126

Symmetry codes: (i) −*x*+1, −*y*+1, −*z*+1; (ii) −*x*+2, *y*+1/2, −*z*+1/2.
